# Using GPS-derived speed patterns for recognition of transport modes in adults

**DOI:** 10.1186/1476-072X-13-40

**Published:** 2014-10-11

**Authors:** Anke Huss, Johan Beekhuizen, Hans Kromhout, Roel Vermeulen

**Affiliations:** Institute for Risk Assessment Sciences, Utrecht University, PO Box 80178, Utrecht, 3508 TD The Netherlands; Institute of Social and Preventive Medicine, University of Bern, Bern, Switzerland; Julius Centre for Public Health Sciences and Primary Care, University Medical Centre, Utrecht, The Netherlands

**Keywords:** Physical activity, Active transport, Motorized, Bike, Walk, Discriminant analysis

## Abstract

**Background:**

Identification of active or sedentary modes of transport is of relevance for studies assessing physical activity or addressing exposure assessment. We assessed in a proof-of-principle study if speed as logged by GPSs could be used to identify modes of transport (walking, bicycling, and motorized transport: car, bus or train).

**Methods:**

12 persons commuting to work walking, bicycling or with motorized transport carried GPSs for two commutes and recorded their mode of transport. We evaluated seven speed metrics: mean, 95^th^ percentile of speed, standard deviation of the mean, rate-of-change, standardized-rate-of-change, acceleration and deceleration. We assessed which speed metric would best identify the transport mode using discriminant analyses. We applied cross validation and calculated agreement (Cohen’s Kappa) between actual and derived modes of transport.

**Results:**

Mode of transport was reliably classified whenever a person used a mode of transport for longer than one minute. Best results were observed when using the 95^th^ percentile of speed, acceleration and deceleration (kappa 0.73). When we combined all motorized traffic into one category, kappa increased to 0.95.

**Conclusions:**

GPS-measured speed enable the identification of modes of transport. Given the current low costs of GPS devices and the built-in capacity of GPS tracking in most smartphones, the use of such devices in large epidemiological studies may facilitate the assessment of physical activity related to transport modes, or improve exposure assessment using automated travel mode detection.

**Electronic supplementary material:**

The online version of this article (doi:10.1186/1476-072X-13-40) contains supplementary material, which is available to authorized users.

## Introduction

Since transport-related physical activity (i.e. walking or bicycling for travel) can contribute considerably to total physical activity, the identification of active or sedentary modes of transport is of relevance e.g. for studies assessing physical activity [1, 2]. Differentiating between modes of transport might also be of interest in studies addressing personal exposure to air pollution [3, 4]. Some studies have used self-reporting, e.g. interviews [5], questionnaires[6] or diaries [7] to assess transport mode, but in recent years other studies have attempted to distinguish modes of transport using either measured accelerometer data [8], combinations of accelerometer data and global positioning system (GPS) data [9], or combinations of GPS with geo-data in a geographic information system (GIS) [10].

GPS receivers have developed into relatively small and low-cost sensors that can be easily used by study participants in epidemiological studies. GPSs have been used for localization purposes, e.g. in combination with accelerometer and geo-data to evaluate the relationship between the built environment and location-based physical activity [11]. Next to position, GPSs also register speed and therefore offer the opportunity to additionally evaluate speed patterns, since speed patterns can differ greatly according to type of transport mode (Additional file 1: Figure S1). Speed alone as measured with GPSs might provide a relatively easy way to differentiate between modes of transport. Previous studies have primarily applied absolute cut-offs to average or maximum trip speeds to identify modes of transport, but have not evaluated which metrics would result in the best classification of transport modes [12, 13]. We assessed which combination of speed metrics would best identify transport modes from GPS data collected under real life conditions.

## Methods

### Data collection

We tracked 12 persons with GPSs during two back- and forth commutes to work at the Institute for Risk Assessment Sciences, Utrecht, the Netherlands, during Summer 2010. Participants were volunteers who were selected based upon their spread in home location and differences in modes of transport (walk, bike, train (but not metro, tram, light rail, subway or similar public transport systems), bus or car) [14]. For the current analysis, we used data collected with an Adapt AD-850 (Adapt-Mobile, London, UK). GPS locations and speed (in km/h) were logged every second, resulting in a total of 215,725 observations. Spatial accuracy was high, with median errors of about 3.5-5 m. Of note, the accuracy of the Adapt AD 850 did not differ materially from two other tested devices [14].

### Data handling

Each speed profile of the commutes was annotated directly using the geographic information system (GIS) ArcGIS 9.3.1 (Esri Redlands, CA, USA), in order to assign true modes of transport (walk/bike/motorized transport in bus, trains or car). Annotation was performed by location based on aerial imagery (Aerodata International Surveys Deurne, Belgium) together with the study participants on their first day of the commute. Because we were more interested in outdoor activities, we removed all logged indoor points (i.e. when being inside of a building, e.g. at home, at work or inside a train station). For each commute, there was a short interval of approximately 10-20s for which we did not know whether the participant was indoors or outdoors. Thus, the exact transition between indoors and outdoors was uncertain and we chose to remove these points. This concerned only a small amount of all logged points (<1.5%).

### Sequences and metrics

We cut each of the speed profiles into separate sequences whenever speed was zero, assuming that zero speed implied no transport and therefore no activity by the participants. Of all sequences, we calculated the duration in seconds, and, in km/h the mean, 95^th^ percentile and standard deviation of speed measurements. We also calculated the rate-of-change metric (RCM) and standardized rate-of-change metric (the RCM divided by the standard deviation over which the RCM is calculated) of speed measurements [15]. The calculation of the RCM is given in Equation 1, where S_n_ is the nth speed measurement in the time series:
1

Within each sequence, we also calculated the differences of consecutive speed observations by subtracting the speed value of the previous observation. Positive values then represent acceleration and negative values deceleration. We then extracted the 95^th^ and the 5^th^ percentile from these differences per sequence, as proxies for high acceleration and deceleration events, respectively.

### Statistical analysis

We applied non-parametric discriminant analysis, based on all combinations of the different speed metrics, including either one, two or three metrics at the same time into the analysis. As metrics we used the mean, 95^th^ percentile and standard deviation of speed, RCM, standardized RCM, acceleration and deceleration in our calculations. To avoid over-fitting, we performed a k-fold cross validation procedure: We split our data sets of 12 persons into two subsets: eight persons to run the discriminant analysis and predict type of activity (“development data set”), and the remaining four persons to calculate a Cohen’s Kappa coefficient between predicted and true type of activity (“validation data set”). For the cross validation, we calculated Cohen’s Kappa coefficients for 152 combinations of groupings of persons: This corresponded to all permutations (n = 495) except those where not all three types of transport mode were included in the development data set (n = 54) or in the validation data set (n = 317). Because sequences had very different durations and since very short sequences would likely be difficult to classify, we compared Kappa values according to groups of sequences with up to 15 seconds, 16–30 seconds, 31–60 seconds and more than a minute duration. This was done in order to assess which time frame would give the most reliable results. We also calculated the observed absolute agreement.

We then combined all train, bus and car commutes into a group of “motorized traffic” and re-ran the analysis. Train, bus and car commutes all represent sedentary travel modes, and this calculation might therefore be of interest if addressing physical activity rather than exposure to e.g. air pollution. Finally, we repeated the analysis including the 441 sequences where all travel modes were included in the development, but not in the validation data set.

All analyses were performed in Stata (version 12, Stata Corp, College Station, Texas, USA).

## Results

**Figure 1:**
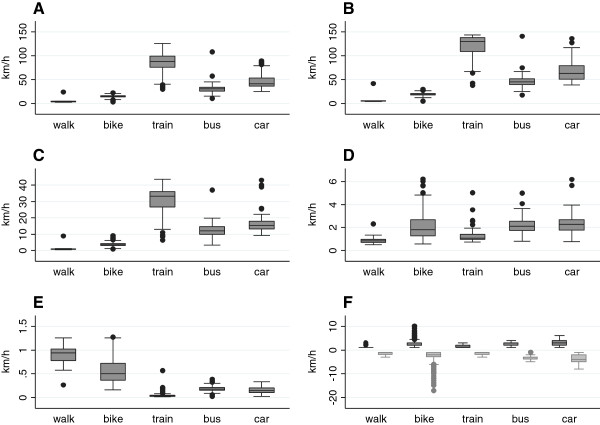
**Speed metrics.** Speed metrics per sequence for each type of transport mode, for sequences of at least one minute duration; Panels **A**: Mean; **B**: 95^th^ percentile of speed; **C**: Standard deviation (sd); **D**: Rate-of-change (RCM); **E**: Standardized rate-of-change (RCMs) metric, **F**: Acceleration (positive values) and deceleration (negative values).

An overview of the number of persons collecting data per mode of transport, the number of sequences and the sequence duration is given in Table 1. Over all sequences, speed appeared to be very low, with walking displaying median speeds of 2, bicycling of 6 and motorized traffic (as a combined category) of about 17 km/h. The average speed increased considerably if speed was calculated based on only those observations that had a least a one minute duration: Median speed increased to about 4, 15 and 48 km/h for walking, bicycling and motorized traffic as a combined category, respectively (Table 1). Speed patterns were quite diverse across the evaluated modes of transport (Figure 1).

**Table 1:** **Number of persons performing a transport mode, speed and duration of sequences**

		All sequences			Sequences of at least one minute duration		
Transport Mode	N	Sequences	Duration	Speed per sequence	Sequences	Duration	Speed per sequence
Walk	2	626	3	2.0	43	94	4.1
			(2–12)	(1.3-3.4)		(70–112)	(3.6-4.7)
Bike	9	1920	7	6.0	327	139	14.9
			(2–30)	(2.0-12.1)		(87–264)	(13.1-16.4)
Train	5	256	6	2.6	77	471	88.3
			(2–185)	(1.5-69.6)		(205–705)	(75.1-104.0)
Bus	3	314	23.	18.9	49	79	31.1
			(5–49)	(6.0-28.5)		(69–101)	(24.1-33.7)
Car	3	131	61	32.9	66	219	41.7
			(22–219)	(11.0-42.0)		(109–387)	(35.6-53.9)

N = Number of subjects, Sequences = number of sequences. Duration is given in seconds, speed in km/h and both duration and speed are listed as medians and interquartile ranges (IQR).

**Figure 2:**
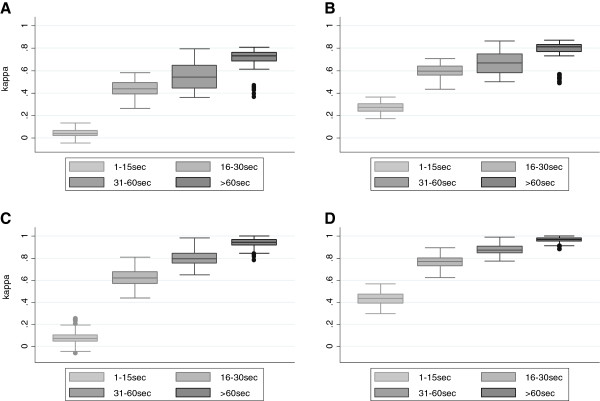
**Kappa coefficients and observed agreement by duration of sequence.** Box plots derived from cross-validation using mean, 95^th^ percentile of speed, standard deviation and deceleration for the discriminant analysis across categories of duration of sequences. Panel **A**: Kappa coefficients classifying walking, bicycling, bus, train and car travels; **B**: Categories as in A, showing proportion agreement; **C**: Kappa coefficients when combining motorized traffic into one category; **D**: Categories as in **C**, showing proportion agreement.

**Figure 3:**
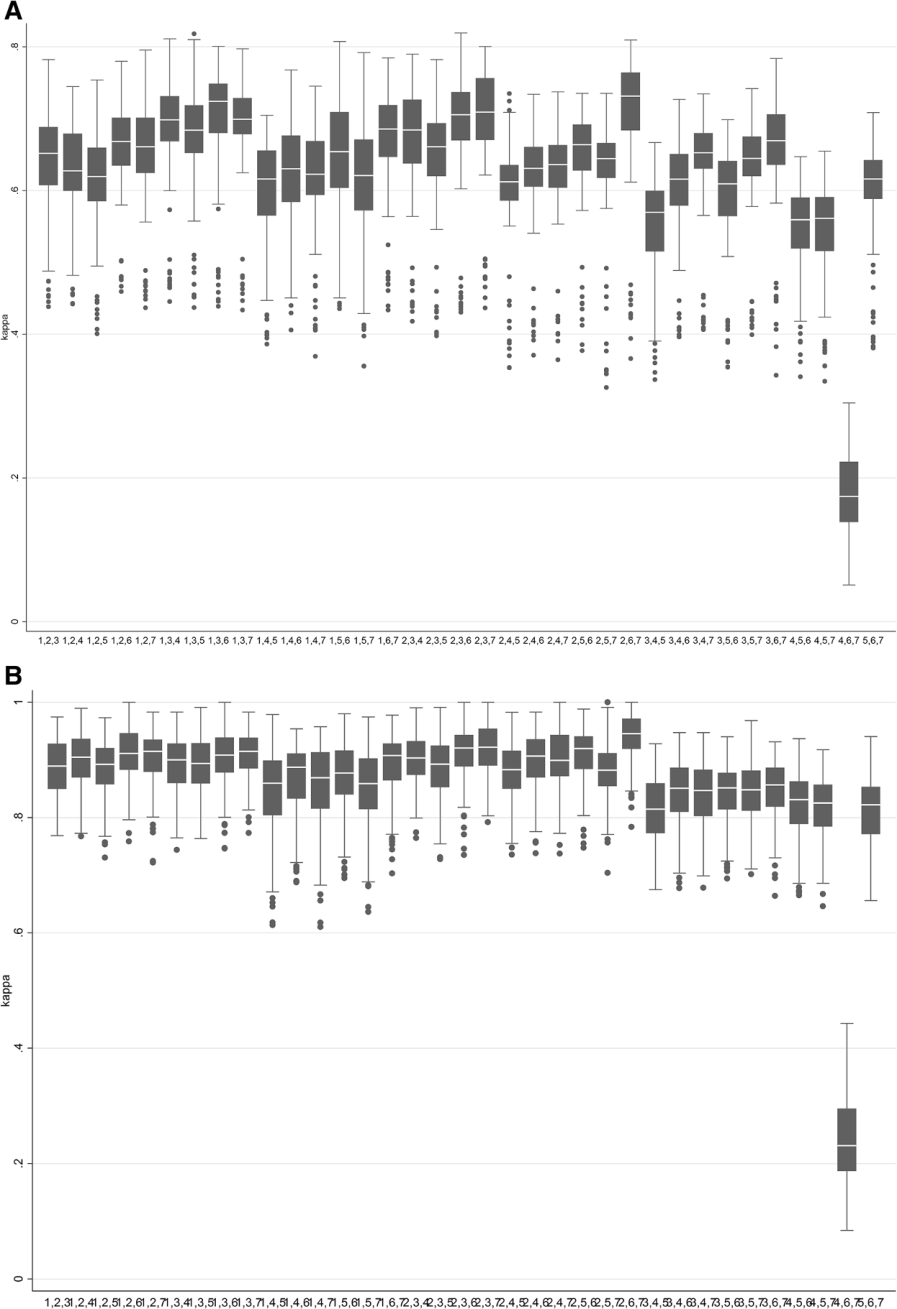
**Results of cross validation using three different speed metrics.** Box plots of Kappa coefficients derived from cross-validation using combinations of different speed metrics in the discriminant analysis, shown are Kappa values for sequences of more than one minute duration. Top panel **(A)**: classifying walking, biking, bus, train and car travels; Bottom panel **(B)**: combining motorized traffic into one category. Legend: 1 = mean, 2 = 95^th^ percentile of speed, 3 = standard deviation, 4 = rate-of-change (RCM), 5 = rate-of-change standardized (RCMs), 6 = acceleration, 7 = deceleration.

Kappa coefficients of the cross-validation runs are shown in Figures 2 and 3. While Kappa coefficients as well as the proportion of observed agreement ranged widely, they increased strongly by increasing duration of the sequences (Figure 2). For sequences of at least one minute duration, discriminant analysis using one metric clearly resulted in highest Kappa coefficients when using the 95th percentile of speed (median Kappa 0.66, IQR 0.65-0.67). When combining two metrics, the highest Kappa coefficients were achieved when using the 95th percentile of speed in combination with either acceleration or deceleration, or when using mean speed and the standard deviation of speed (all median Kappas 0.67, IQR 0.66-0.68; Additional file 1: Figure S2). When using three metrics, Kappa was highest when combining the 95th percentile with acceleration and deceleration (median Kappa 0.73, IQR 0.72-0.74); such Kappas can be interpreted as “substantial agreement” [16, 17]. All other combinations of speed metrics included in the discriminant analysis resulted in lower Kappa values (Figure 3).

Kappas increased to values above 0.9 if bus, train and car commutes were combined into one category of motorized transport: The highest Kappa was again obtained when using the 95^th^ percentile of speed in combination with acceleration and deceleration (Kappa 0.95, IQR 0.94-0.95). These Kappas can be interpreted as “excellent” or to represent “almost perfect agreement” [16, 17].

When we included those sequences where all different types of transport modes were included in the development, but not in the validation data set, this resulted in a few low outliers of the Kappa values. Median or interquartile ranges were, however, not affected (data not shown). This was due to cross-validation runs that included only one or two activities, resulting in high observed and expected agreement. For example, one combination where only bicycling was included in the validation data set resulted in a Kappa of zero, although observed agreement was 97%.

The vast majority of all sequences were very short and displayed low Kappa coefficients. However, given the average duration of 252 seconds of all sequences longer than at least one minute, this translates into 83% of all observation-time that could be classified with high reliability.

## Discussion

Speed profiles as recorded from GPS devices can be used to differentiate between the modes of transport of study participants. While for very short-duration sequences the transport modes were difficult to identify, activities lasting for at least one minute could be reliably classified. The majority of sequences were relatively short, but because longer sequences of more than one minute duration contributed more to total observation-time, over 80% of all observations could be assigned the correct mode of transport with high accuracy.

A strength of our study is that we had a reasonably large amount of observations (>200.000 logged GPS positions) that we could analyze and that were collected under real-life conditions. A second strength is that the calculation was based on only a few different speed metrics as logged by a GPS, which makes this procedure a likely feasible approach also for larger study populations, if GPS data is available. Thirdly, the true mode of transport was assigned together with the study participants based on location and aerial imagery. In order to circumvent potential problems of recalling the travelled route, annotation of the route was performed on the day of the commute. Since volunteers also were very familiar with the routes they usually travelled on working days, we are confident that this procedure resulted in high-quality validation data.

A weakness of our study includes that only 12 persons collected data and that within persons, sequences (and travels) were not independent, although we treated them as such in our analysis. We performed our validation across persons to counteract this, but our Kappas might nevertheless have been affected. It is, however, unclear if this would lead to an upward- or downward shift of the Kappas. Another disadvantage is that our data collection was performed in a very flat area. Speed profiles may be more variable in topographically more challenging areas. In addition, speed profiles of motorized traffic may also be more difficult to disentangle from bicycling if driving is restricted to urban areas with dense traffic and slower driving speeds. Cycling may also display quite similar speed profiles as jogging/running, although jogging/running is rarely used for commuting and would be therefore less of an issue when assessing modes of transport. Only two persons contributed to our walking data set. Although a small data set, the speeds are very close to those previously reported in the scientific literature [18]. Reference values for walking speeds have been reported to lie between about 3.3-5.1 km/h [19]. Also, given the strong differences in speed profiles to the next faster category (bicycling, with about 15 km/h), even a larger data set capturing a higher variability in walking profiles would be unlikely to result in deteriorated recognition of this transport mode. Walking has also previously been reported to be captured with high reliability from speed data alone, because walking displays consistently low speeds during trips [20, 21]. Finally, our study group was restricted to a relatively homogenous group of employees (age group 25–60 years), and speed patterns may therefore not be generalizable to elderly persons or to children, the latter for instance, have been described to display a much more erratic movement behavior [22].

Alternative methods to assess modes of transport include for example diary data recorded by study participants, but this type of data collection requires relatively high levels of commitment of participants, especially if data collection is performed over several days. Adding accelerometer data could help in further separating activities that display similar speed profiles, such as bicycling and inline skating. Accelerometer data provides the additional opportunity to evaluate energy expenditure. However, applying two different devices, while increasing the amount of information, will on the other hand increase costs through an increased workload to collect and process the data. Also, the amount of data collected with triaxial accelerometers can be huge, depending on the time resolution, and might pose some additional challenges. The combination with other sensor data, such as from pedometers, the gyroscope or from heart rate monitors may further improve transport mode detection. If available, adding contextual location information (e.g. location of tram, bus or train stations to GPS-logged locations) in a geographic information system will likely enable to improve travel mode detection even further. This is because information on environmental features can be added, leading e.g. to the possibility to add non-logged positions (e.g. when taking an underground) [13].

In summary, discriminant analysis based on a few speed metrics showed that modes of transport can be reliably assessed from GPS data alone. Using the 95^th^ percentile of speed in combination with acceleration and deceleration provided best results.

### Perspectives

Active transport to school or work can contribute to total physical activity levels [23, 24]. Objective measurements performed with GPSs would be expected to contribute to more accurate quantification of transport-related levels of physical activity, compared with, for example, self-report. Improving the assessment of physical activity levels would in turn contribute to a more accurate assessment of the association between physical activity and health effects and the design of effective interventions [25]. The ability to differentiate transport modes from speed data alone may be of use in future large epidemiological studies assessing transport-related physical activity or traffic related exposures.

Currently, smartphones have integrated sensors that include among others GPS receivers, and applications have been developed that can evaluate transport modes. However, the application of this technology for larger epidemiological studies may still be hampered in that applications are not necessarily freely available, privacy issues may arise, and there may be comparability issues between devices. Constant GPS activation and logging considerably drains mobile phone batteries. Methods such as variable rate logging have been developed to increase battery time, [26] but so far this issue still remains a limiting factor for the application into wider population studies.

## Electronic supplementary material

Additional file 1: Figure S1: Example of speed profiles of different transport modes. **Figure S2.** Results of cross validation using one or two speed metrics at a time. (PDF 62 KB)
