# Supplementary material for: Using GPS-derived speed patterns for recognition of transport modes in adults
Source: Int J Health Geogr. 2014 Oct 11;13:40. doi: 10.1186/1476-072X-13-40 (PMC4320483; doi:10.1186/1476-072X-13-40)
Supplement: Supplementary file 1 — Additional file 1: Figure S1: Example of speed profiles of different transport modes. Figure S2. Results of cross validation using one or two speed metrics at a time. (PDF 62 KB) [file 12942_2014_621_MOESM1_ESM.pdf]

Supplementary Figure 1: Example of speed profiles of different transport modes

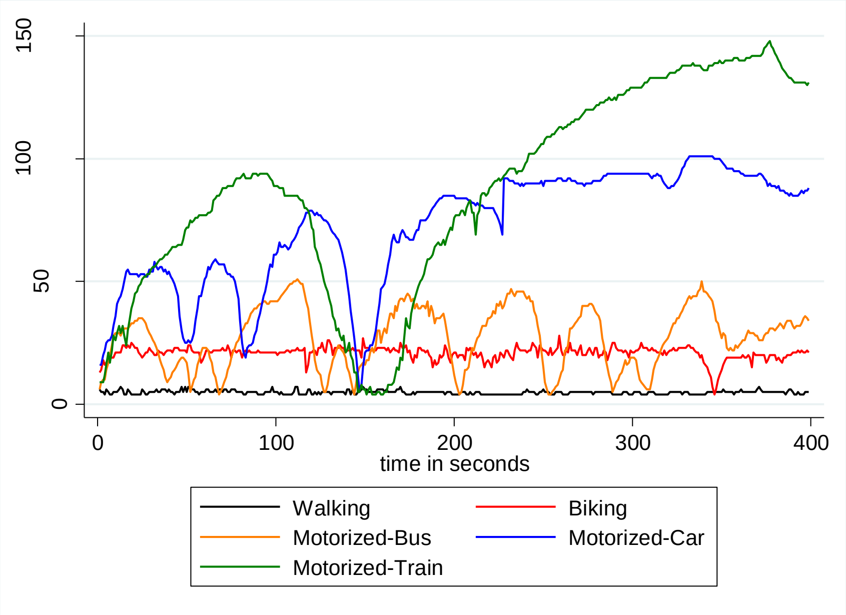

Supplementary Figure 2: Results of cross validation using one or two speed metrics at a time

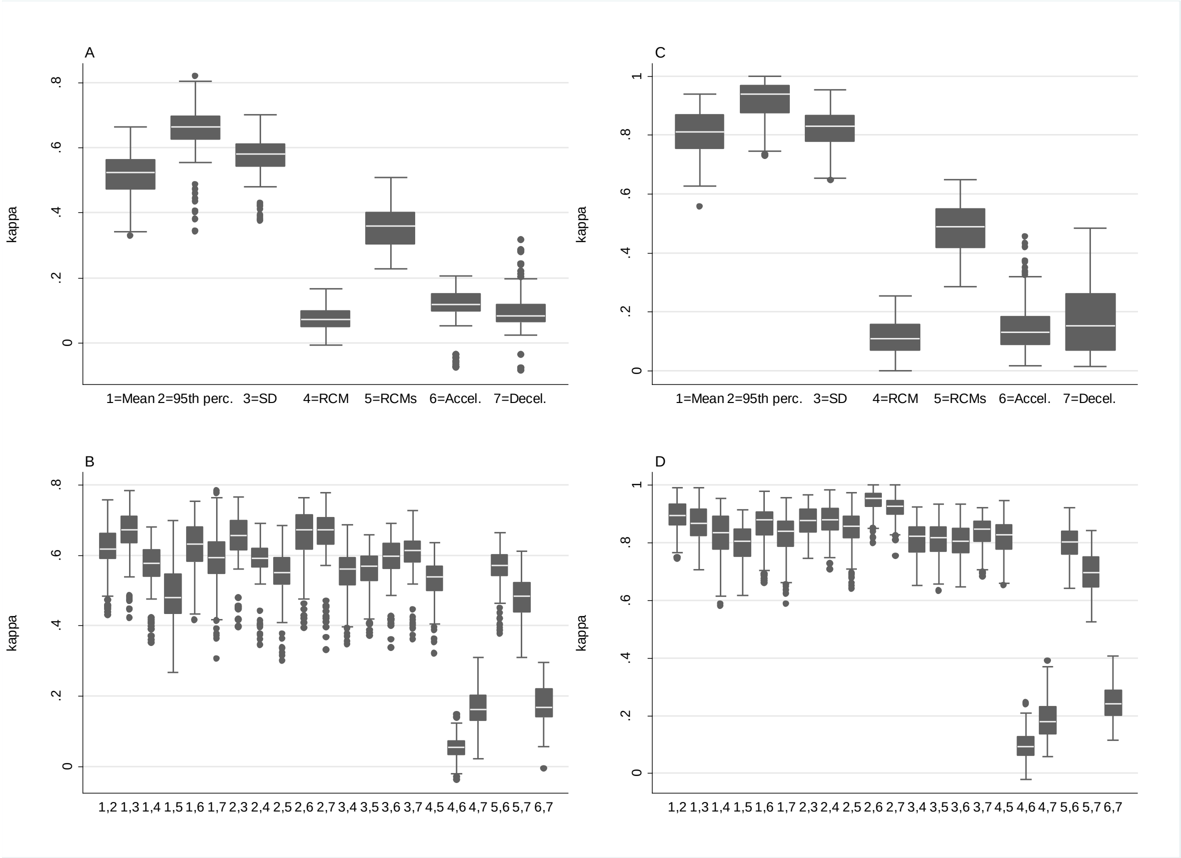

Box plots of Kappa coefficients derived from cross-validation using combinations of different speed metrics in the discriminant analysis, shown are Kappa values for sequences of more than one minute duration. Panels A: One metric, classifying walking, biking, bus, train and car travels; B: One metric, summarizing motorized traffic in one category. C: Two metrics, classifying walking, biking, bus, train and car travels; D: Two metrics, combining motorized traffic into one category. Legend: 1=mean, 2=95th percentile of speed, 3=standard deviation, 4=rate-of-change (RCM), 5=rate-of-change standardized (RCMs), 6=acceleration, 7=deceleration
